# A unique hybrid-structured surface produced by rapid electrochemical anodization enhances bio-corrosion resistance and bone cell responses of β-type Ti-24Nb-4Zr-8Sn alloy

**DOI:** 10.1038/s41598-018-24590-x

**Published:** 2018-04-26

**Authors:** Chia-Fei Liu, Tzu-Hsin Lee, Jeng-Fen Liu, Wen-Tao Hou, Shu-Jun Li, Yu-Lin Hao, Haobo Pan, Her-Hsiung Huang

**Affiliations:** 1 Institute of Oral Biology, National Yang-Ming University, Taipei, Taiwan; 2 Department of Dentistry, Changhua Christian Hospital, Changhua, Taiwan; 3 Department of Stomatology, Taichung Veterans General Hospital, Taichung, Taiwan; 4 Shenyang National Laboratory for Materials Science, Institute of Metal Research, Chinese Academy of Sciences, Shenyang, China; 5 Center for Human Tissues and Organs Degeneration, Shenzhen Institute of Advanced Technology, Chinese Academy of Sciences, Shenzhen, China; 6 Department of Dentistry, National Yang-Ming University, Taipei, Taiwan; 7 Graduate Institute of Basic Medical Science, China Medical University, Taichung, Taiwan; 8 Department of Medical Research, China Medical University Hospital, Taichung, Taiwan; 9 Department of Bioinformatics and Medical Engineering, Asia University, Taichung, Taiwan; 10 Department of Stomatology, Taipei Veterans General Hospital, Taipei, Taiwan; 11 Department of Education and Research, Taipei City Hospital, Taipei, Taiwan

## Abstract

Ti-24Nb-4Zr-8Sn (Ti2448), a new β-type Ti alloy, consists of nontoxic elements and exhibits a low uniaxial tensile elastic modulus of approximately 45 GPa for biomedical implant applications. Nevertheless, the bio-corrosion resistance and biocompatibility of Ti2448 alloys must be improved for long-term clinical use. In this study, a rapid electrochemical anodization treatment was used on Ti2448 alloys to enhance the bio-corrosion resistance and bone cell responses by altering the surface characteristics. The proposed anodization process produces a unique hybrid oxide layer (thickness 50–120 nm) comprising a mesoporous outer section and a dense inner section. Experiment results show that the dense inner section enhances the bio-corrosion resistance. Moreover, the mesoporous surface topography, which is on a similar scale as various biological species, improves the wettability, protein adsorption, focal adhesion complex formation and bone cell differentiation. Outside-in signals can be triggered through the interaction of integrins with the mesoporous topography to form the focal adhesion complex and to further induce osteogenic differentiation pathway. These results demonstrate that the proposed electrochemical anodization process for Ti2448 alloys with a low uniaxial tensile elastic modulus has the potential for biomedical implant applications.

## Introduction

Millions bone graft procedures are expected to be performed annually to fill bone defects or to improve fracture healing and repair. This number is expected to continue to increase with the rapid growth of the elderly population. Therefore, the development of a suitable material for repairing and regenerating bones that have been fractured due to disease, trauma and aging is a significant clinical challenge^1^. Titanium (Ti) and Ti-based alloys are widely used as biomaterials for orthopedic and dental implant applications due to their suitable mechanical properties, corrosion resistance and biocompatibility among all metallic biomaterials^2,3^. Although Ti and Ti-based alloys have specific and excellent properties for biomedical applications, some issues must be addressed for the long-term implantation of these materials. One of the important issues is biomechanical incompatibility: the difference in elastic modulus between the biomedical Ti metals and the natural bone can result in bone resorption because of the stress-shielding effect^4,5^.

Compared with conventional metallic materials, Ti and Ti-based alloys show a relatively lower elastic modulus than those of stainless steel (200 GPa) and Co-Cr-Mo alloys (200–230 GPa). However, the elastic moduli of the commonly used commercial Ti and Ti-based alloys (100–110 GPa)^6^, such as commercially pure Ti (CP-Ti), Ti-6Al-4V and Ti-6Al-7Nb, are still an order of magnitude greater than that of human cortical bone (10–30 GPa)^7,8^. This mismatch between the elastic modulus of bone and that of the implant will cause an insufficient load transfer from the implant to the adjacent bone and, thus, induce a stress-shielding effect at the sites of load-bearing bones during long-term implantation. This type of stress-shielding effect will lead to bone resorption at the bone-implant interface and eventually result in implant failure^9,10^.

β-type Ti alloys have been the most attractive materials for overcoming the elastic modulus incompatibility between the bone and Ti-based implants for orthopedic applications due to their low elastic moduli^3,11,12^. Of all β-type Ti alloys, Ti-24Nb-4Zr-8Sn (wt%, hereafter designated Ti2448) is a recently developed β-type Ti alloy for biomedical applications. This novel alloy consists of biocompatible elements and possesses a low uniaxial tensile elastic modulus of approximately 45 GPa^13,14^, which is close to that of human cortical bone. Such a low elastic modulus may prevent the stress-shielding effect caused by the inhomogeneous stress transfer between a metal implant and the adjacent bone. Furthermore, this alloy not only has a low elastic modulus close to that of human bone but also has high mechanical strength, which is an ideal combination of mechanical properties not available in the other β-type Ti alloys.

For long-term clinical use, the corrosion resistance of metallic materials is a great concern especially when materials are implanted in the virulent electrolytic environment of the human body because corrosion leads to gradual degradation of materials by electrochemical attack. Therefore, the corrosion performance of Ti2448 is extremely important for orthopedic and dental implant applications. Cheng *et al*. reported that for dental implant applications, the Ti2448 alloy presents a corrosion resistance similar to that of CP-Ti and Ti6Al4V alloy in simulated oral environments^15^. Bai *et al*. found that the corrosion resistance of the Ti2448 alloy is better than that of Ti6Al4V alloy but comparable to that of CP-Ti in different simulated physiological solutions^16,17^. It has been commonly accepted that Ti and Ti-based alloys show exceptional corrosion resistance due to the natural formation of a passive oxide layer. However, this protective passive layer, which inhibits the release of metal ions, may become unstable in the human body due to deterioration during long-term implantation. Once the passive surface layer is disrupted, corrosion proceeds and metal ions are released continuously, which leads to ions accumulation and, thus, may result in potentially biological side effects, depending on the species and concentrations of ions, and eventually implant failure^18,19^. Although the corrosion behavior of the Ti2448 alloy is similar to that of Ti, there is still a potential risk of metal ion release from the Ti2448 alloy due to the corrosion process. Therefore, determining how to further improve the bio-corrosion resistance of the Ti2448 alloy for future long-term implantation is one of the important purposes of this study.

Moreover, the surface oxide layer of metallic materials plays an influential role not only in corrosion resistance but also in the biocompatibility of materials. It is well known that surface properties, such as surface topography, roughness, chemical composition and wettability, are important factors in biocompatibility because they affect the biological responses at the bone-implant interface^20–22^. Surface topography is a key surface property because it is able to regulate the cell responses to material surfaces. Some studies have reported the design and creation of surface geometries with suitable nanoscale topography can improve cell responses, such as cell adhesion^23^, migration^24^, proliferation^25^ and differentiation^26^. There are various surface treatments that can create nanoscale topography on metallic materials to provide the desired biological responses. In our previous studies, the electrochemical anodization treatment is used to produce a nano-networked oxide layer on the surfaces of CP-Ti and Ti-based alloys to improve the bio-corrosion resistance, hemocompatibility and cell responses^27–29^.

Our hypothesis is that a hybrid surface structure, featuring a mesoporous outer topography and a dense inner section, can be produced on the surface of the new β-type Ti2448 alloy using a simple and rapid electrochemical anodization treatment to improve the bio-corrosion resistance and bone cell responses. To analyze this hypothesis, polished Ti2448 specimens were created, as well as polished Ti control specimens, and the Ti2448 specimens were treated with an electrochemical anodization treatment in alkaline solution to produce a hybrid oxide layer. Within this research, the surface characteristics, such as the surface morphology, oxide layer thickness, crystal structure and wettability, were investigated after the anodization treatment. Furthermore, the bio-corrosion behaviors of the anodized Ti2448 alloys in simulated body environments were evaluated. To provide helpful indicators for further *in vivo* studies and clinical applications, various biological responses, such as the protein adsorption and the cell adhesion, migration, proliferation, mineralization and differentiation, of the anodized Ti2448 alloy were studied.

## Results

### Surface characteristics

**Figure 1:**
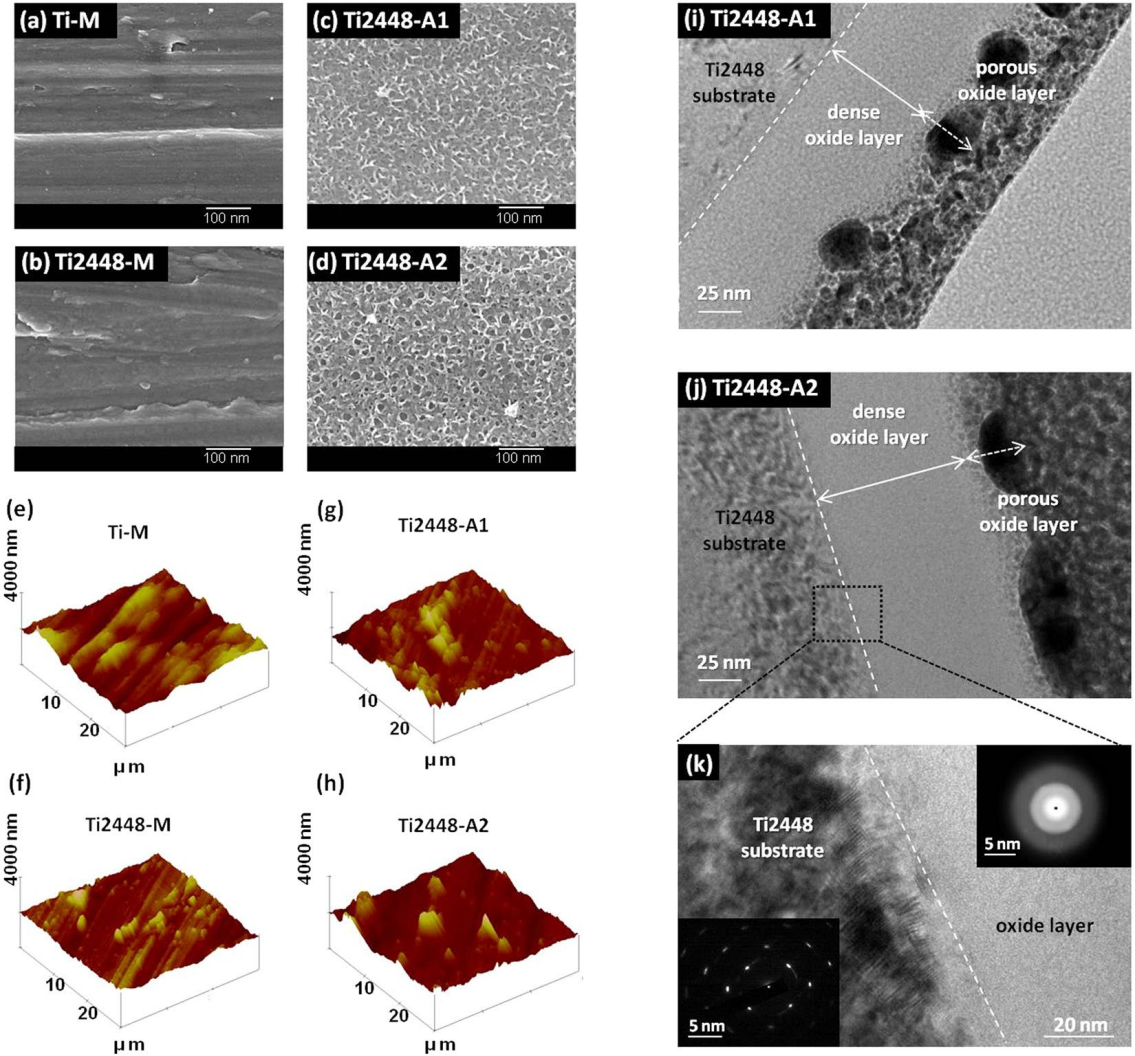
Surface FE-SEM morphologies of the test Ti and Ti2448 specimens. (**a**) Ti-M: Ti specimen mechanically polished with SiC paper up to #1200; (**b**) Ti2448-M: Ti2448 specimen mechanically polished with SiC paper up to #1200; (**c**) Ti2448-A1: Ti2448-M treated through electrochemical anodization with current A1; (**d**) Ti2448-A2: Ti2448-M treated with current A2. Surface AFM topography and roughness of the test Ti and Ti2448 specimens: (**e**) Ti-M; (**f**) Ti2448-M; (**g**) Ti2448-A1; (**h**) Ti2448-A2. Cross-sectional TEM images of the anodized Ti2448 specimens: (**i**) Ti2448-A1; (**j**) Ti2448-A2; (**k**) Higher magnification of (**j**) with the diffraction patterns of the selected area.

The microscopic (nanometer) scale surface morphologies of Ti2448 before and after the electrochemical anodization treatment are shown in Fig. 1(a–d). The FE-SEM images, covering an image area of approximately 450 nm × 600 nm, of the mechanically ground Ti (Ti-M) and the Ti2448 (Ti2448-M) only showed oriented ground grooves (Fig. 1(a) and (b)). After the electrochemical anodization treatment, a mesoscale porous topography was formed on the anodized Ti2448 (Ti2448-A1 and Ti2448-A2) surface (Fig. 1(c) and (d)). The pore size and distribution of anodized Ti2448 specimens are shown in Fig. S1. The pore sizes of the porous oxide layer on the Ti2448-A1 and Ti2448-A2 mainly ranged from a few nm to approximately 50 nm. The pore sizes of Ti2448-A1 located mostly on 10–30 nm (over 10% occurrence rate in Fig. S1(a)); however, the pore sizes of Ti2448-A2 treated with a larger anodic current for a longer time, located mostly on 20–40 nm (over 10% occurrence rate in Fig. S1(b)). Based on the results shown in Fig. 1(c) and (d) and Fig. S1, Ti2448-A2 showed a larger pore size than Ti2448-A1.

AFM was used to evaluate the effect of the anodization treatment on the macroscopic (micrometer) scale surface roughness (R_a_) of Ti2448-A1 and Ti2448-A2 with a scanning area of 30 μm × 30 μm (Fig. 1(e–h)). The evaluation showed that there were no significant differences in R_a_ between the treated and untreated test specimens. For all test specimens, the R_a_ was approximately 0.1 µm.

Furthermore, the thickness and crystal structure of the hybrid oxide layer on Ti2448-A1 and Ti2448-A2 were analyzed by TEM and are also shown in Fig. 1(i–k). The TEM images indicated that the cross-sectional thickness of the hybrid oxide layer on Ti2448-A1 and Ti2448-A2 was approximately 50 to 80 nm and 70 to 120 nm, respectively (Fig. 1(i) and (j)). Compared with the untreated Ti-M and Ti2448-M, the treated Ti2448-A1 and Ti2448-A2 exhibited thickening of the surface oxide layer. The crystal structure of the oxide layer was amorphous (Fig. 1(k)). The oxide layer could be separated into two different sections: the outer porous section close to the surface and the inner nonporous section between the substrate and the outer porous section. Compared with the outer section, the inner section had a structure that was tighter with a greater density (Fig. 1(i) and (j)).

XPS depth profiling data, in terms of the atomic concentration (%) of O, Ti, Nb, Zr and Sn elements, of the test specimens’ surfaces are shown in Fig. S2. For both Ti2448-A1 and Ti2448-A2 specimens, the amounts of the above-mentioned elements in the outer (etching time for 20 sec) and inner (etching time more than 200 sec) oxide layers were in the order: O > Ti > Nb > Sn > Zr (outer layer) and O > Ti > Nb > Zr > Sn (inner layer), respectively. The oxides corresponding to Ti, Nb, Zr and Sn elements on the anodized Ti2448 alloys were mainly TiO_2_, Nb_2_O_5_, ZrO_2_ and SnO, respectively. Therefore, the oxides in either outer or inner oxide layer on both Ti2448-A1 and Ti2448-A2 specimens contained mainly TiO_2_ and Nb_2_O_5_ with small amounts of SnO and ZrO_2_.

**Figure 2:**
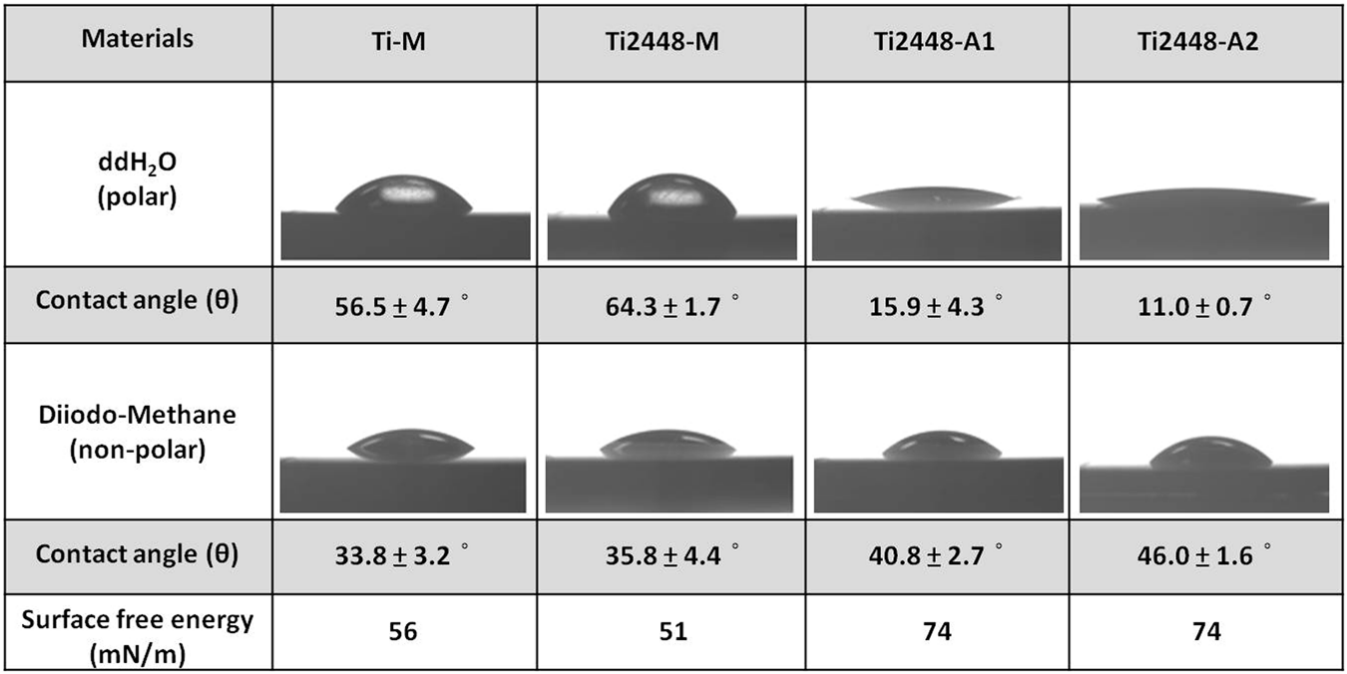
Contact angle and surface free energy of the test Ti and Ti2448 specimens.

Finally, a contact angle goniometer was used to image and calculate the contact angle and the surface free energy to evaluate the wettability of the test specimens. The result shown in Fig. 2 indicated that the polar double-distilled H_2_O on the anodized Ti2448-A1 and Ti2448-A2 showed a smaller contact angle (11–16°) than the contact angle on the untreated Ti-M and Ti2448-M (> 56°). In contrast, the surface free energies of Ti2448-A1 and Ti2448-A2 were much greater than the surface free energies of the untreated groups, suggesting that Ti2448-A1 and Ti2448-A2 exhibited better surface wettability than Ti-M and Ti2448-M.

The above-mentioned results indicate that the electrochemical anodization treatment did not change the macroscopic surface roughness on a µm scale, but successfully created a hydrophilic hybrid oxide layer that consisted of an outer mesoporous section and an inner dense section.

### Bio-corrosion resistance

**Figure 3:**
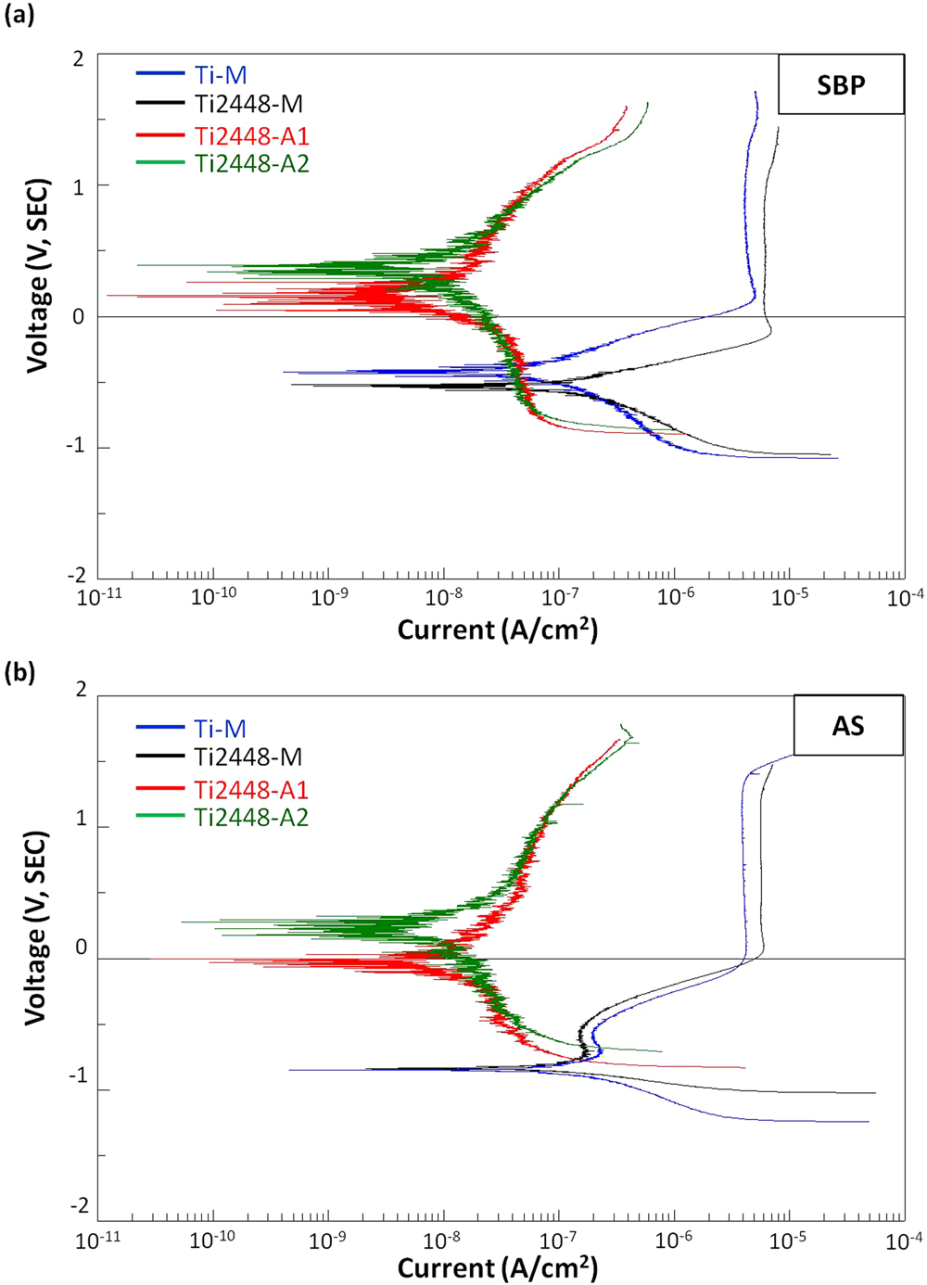
Polarization curves of the test Ti and Ti2448 specimens in different electrolytes: (**a**) SBP; (**b**) AS.

The polarization curves for Ti-M, Ti2448-M, Ti2448-A1 and Ti2448-A2 in the SBP and AS solutions are shown in Fig. 3. Compared to the corrosion potential (E_corr_) of the untreated Ti-M and Ti2448-M, the E_corr_ of Ti2448-A1 and Ti2448-A2 shifted to much nobler values. In addition, it is clear indication that the anodic and cathodic current densities of the anodized specimens were far lower than that of the untreated specimens. In the SBP solution, the values of the corrosion rate (I_corr_) for Ti-M, Ti2448-M, Ti2448-A1 and Ti2448-A2 were 0.041, 0.102, 0.003 and 0.004 μA/cm^2^, respectively. In the AS solution, the I_corr_ values of Ti-M, Ti2448-M, Ti2448-A1 and Ti2448-A2 were 0.028, 0.093, 0.007 and 0.010 μA/cm^2^, respectively. In both the SBP and AS solutions, the I_corr_ of the anodized Ti2448 group was less than that of the untreated Ti-M and Ti2448-M. The above-mentioned results suggest that the corrosion resistance of Ti2448-A1 and Ti2448-A2 was better than that of Ti2448-M, indicating that the hybrid oxide layer produced by the electrochemical anodization treatment provided better protection than the surface of the untreated group.

### Protein adsorption ability

**Figure 4:**
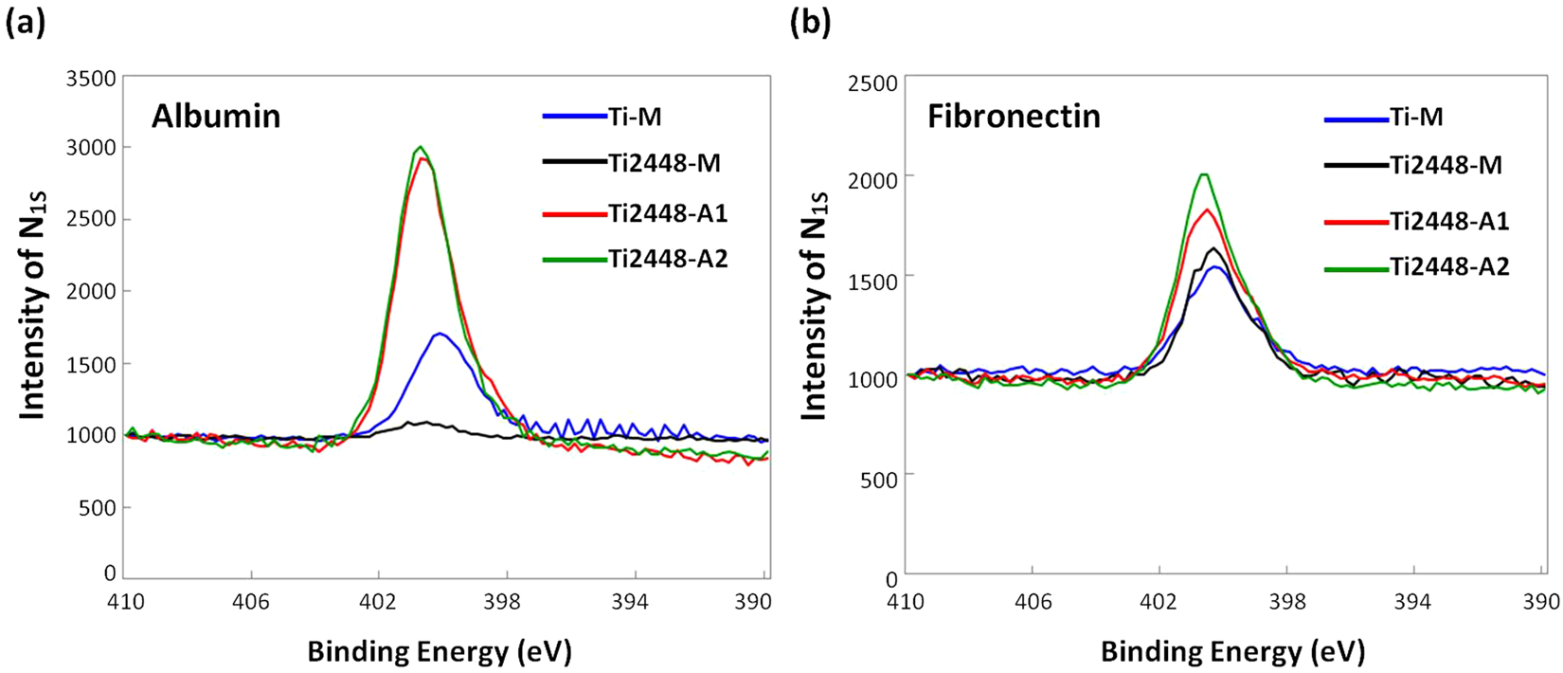
Protein adsorption analysis, in terms of the N1s spectra, obtained using an XPS on the test Ti and Ti2448 specimens: (**a**) albumin; (**b**) fibronectin.

Protein adsorbed on the surface of material is the very beginning step before cells attachment. Moreover, it might affect the following cell responses especially cell adhesion. Therefore, the protein adsorption, in terms of nitrogen (N1s) on all test specimens was measured using XPS and was shown in Fig. 4. The result indicated that the treated Ti2448-A1 and Ti2448-A2 showed higher N1s intensities than the untreated Ti-M and Ti2448-M in both the albumin (Fig. 4(a)) and fibronectin (Fig. 4(b)) solutions. The amount of albumin and fibronectin adsorbed on the Ti2448-A1 and Ti2448-A2 was more than 1–3 times than that on the untreated Ti-M and Ti2448-M, indicating that the electrochemical anodized treatment could improve the protein adsorption ability of Ti2448 alloy.

### *In vitro* hMSCs cell responses

**Figure 5:**
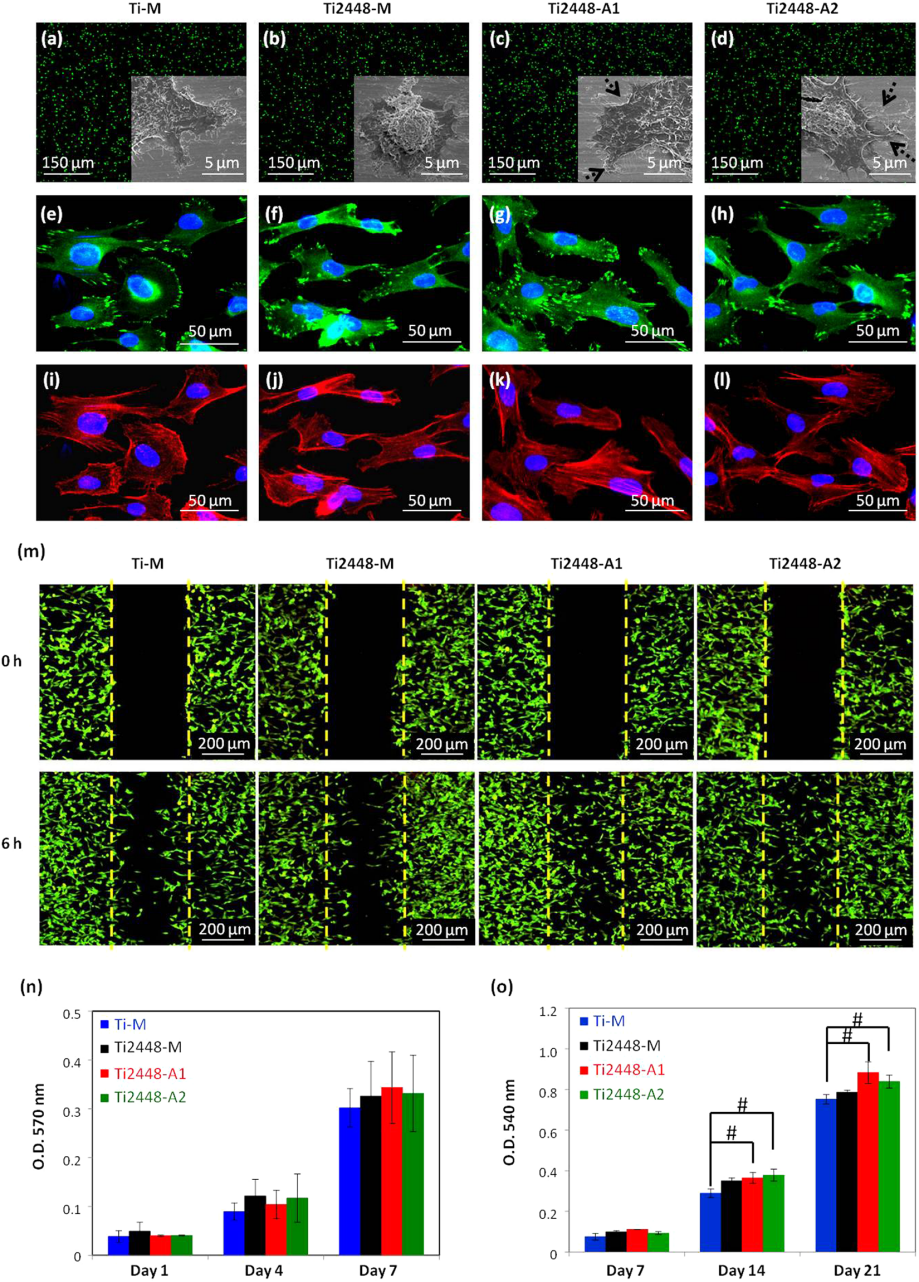
Cell distribution and adhesion morphologies, observed using fluorescence microscopy and FE-SEM, of hMSCs-GFP cultured on the test Ti and Ti2448 specimens for 1 h: (**a**) Ti-M; (**b**) Ti2448-M; (**c**) Ti2448-A1; (**d**) Ti2448-A2. Immunofluorescence images of focal adhesion complex formation and cytoskeletal arrangement, observed using fluorescence microscopy, of the hMSCs cultured on the test Ti and Ti2448 specimens for 6 h: (**e**,**i**) Ti-M; (**f**,**j**) Ti2448-M; (**g**,**k**) Ti2448-A1; (**h**,**l**) Ti2448-A2 (green: vinculin; red: F-actin; blue: nucleus). (**m**) Cell migration, observed using fluorescence microscopy, of the hMSCs-GFP on the test Ti and Ti2448 specimens. (**n**) Cell proliferation and (**o**) cell mineralization analyzed using MTT assay and Alizarin Red S staining, respectively, of the hMSCs on the test Ti and Ti2448 specimens. Data are shown as the mean ± SD. (^#^p < 0.05 indicates a statistically significant difference compared to Ti-M).

The adhesion of osteogenic cells on biomaterials is the initial and an important step of cell-material interactions. Cell attachment and spreading occur in the early stage of cell adhesion on materials. Therefore, in this study, hMSCs-GFP were seeded on the test specimens and incubated for 1 h to observe the *in situ* cell distribution, cell membrane extension morphology and cell spreading area, by fluorescence microscope and FE-SEM (Figs 5(a–d) and S3(a)). As shown in Fig. 5(a–d), the fluorescence images showed similar cell distribution among the test specimens. In contrast, compared with the cells cultured on the untreated Ti-M and Ti2448-M, the cells cultured on the anodized Ti2448-A1 and Ti2448-A2 showed cell membrane extension morphology and spreading area similar to that seen on Ti-M, and better than that seen on Ti2448-M (Figs 5(a–d) and S3(a)).

The expression of focal adhesion complex proteins and the development of the cytoskeletal arrangement occur during the later stage of the adhesion of cells to materials and induce signal transduction to regulate the subsequent cell responses. Therefore, the immunofluorescence staining was used to observe the expression of one of focal adhesion-related protein, vinculin, and F-actin arrangement, which were used as indicators for the focal adhesion complex formation and the alteration in the cytoskeletal arrangement. The results showed that the hMSCs cultured on the anodized Ti2448-A1 and Ti2448-A2 for 6 h exhibited greater focal adhesion complex formation and better cytoskeletal arrangement compared with the cells on the untreated Ti-M and Ti2448-M (Figs 5(e–l) and S3(b)).

Cell migration and proliferation are two important steps in the bone repair process; thus, it is important to observe both these processes on the test specimens. In this study, wound-healing assay and MTT assay were used to estimate the effect of each of the test specimens (Ti-M, Ti2448-M, Ti2448-A1 and Ti2448-A2) on the migration and proliferation, respectively. As shown in Fig. 5(m), compared with the cells on the untreated Ti-M and Ti2448-M, more of the cells that were cultured on the anodized Ti2448-A1 and Ti2448-A2 directionally migrated toward the wound. Moreover, the migration speed on Ti2448-A1 and Ti2448-A2 was approximately 2-fold faster than that on Ti-M and Ti2448-M. This result indicated that the anodized Ti2448-A1 and Ti2448-A2 could increase the mobility of cells compared with the untreated Ti-M and Ti2448-M. Figure 5(n) showed the proliferation results which indicating that there was no significant differences in the number of cells on the various test specimens during the 7 days of incubation.

Cell differentiation is another essential step in the bone repair and osteointegration process. To estimate the capacity for mesenchymal stem cells to differentiate into osteogenic lineages, cell mineralization, as determined by Alizarin Red S staining, was used as an effective indicator of the level of cell differentiation, and the result was shown in Fig. 5(o). After 14 and 21 days of osteogenic incubation, the hMSCs cultured on the anodized Ti2448-A1 and Ti2448-A2 showed statistically significantly better cell mineralization than those on Ti-M. This result indicated that the electrochemical anodization treatment could induce both extracellular matrix mineralization of hMSCs and more calcium deposition on Ti2448-A1 and Ti2448-A2 than on Ti-M and Ti2448-M after 14 and 21 days of osteogenic incubation.

**Figure 6:**
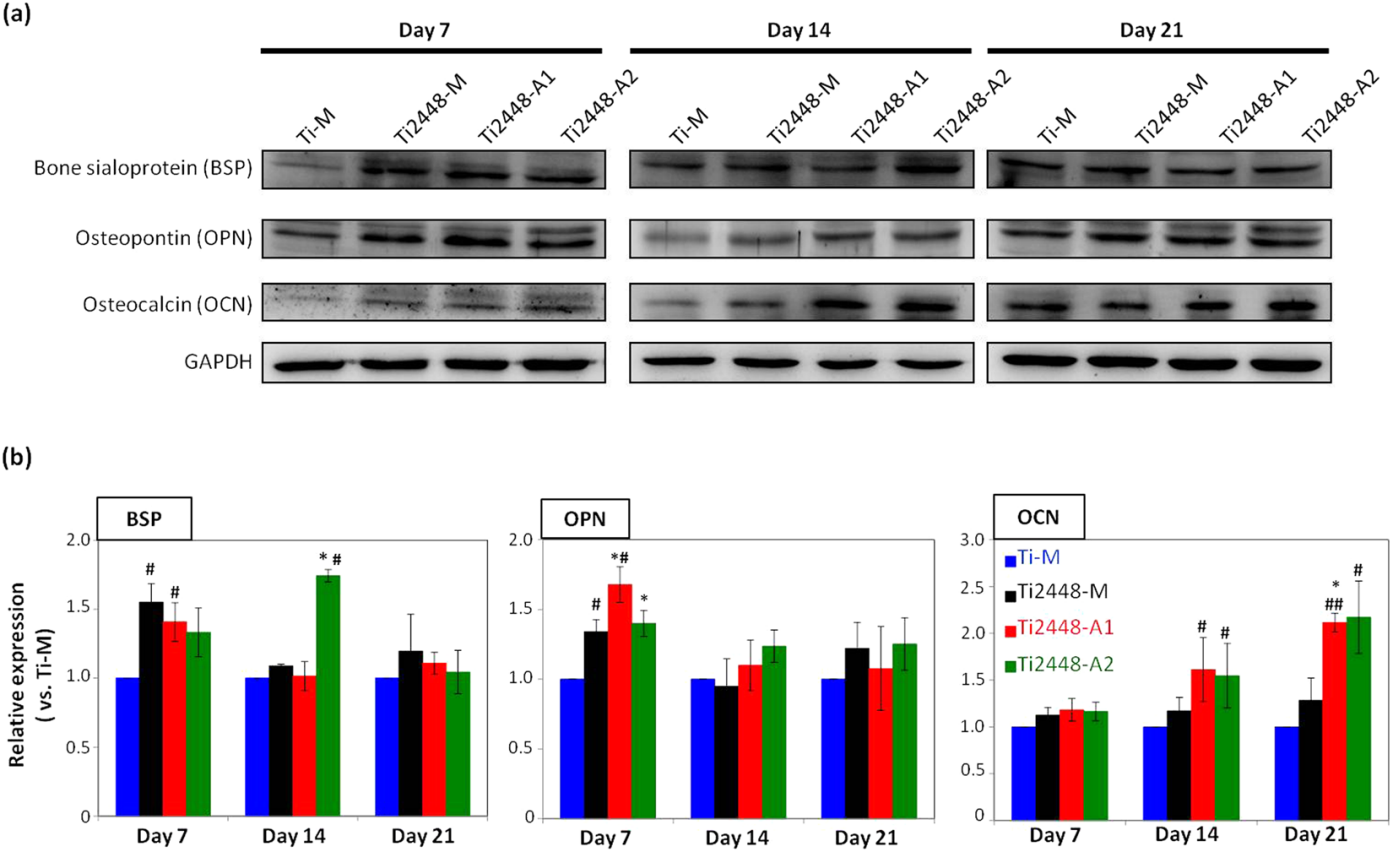
Osteogenic protein marker expression, assessed using Western blotting analysis, of the hMSCs on the test Ti and Ti2448 specimens: (**a**) Representative electrophoresis images of osteogenic-related protein expression after incubation of 7, 14 and 21 days; (**b**) the quantitative levels of osteogenic-related protein expression in (**a**). The data are shown as the mean ± SD. (*p < 0.05 indicates a statistically significant difference compared to Ti2448-M; ^#^p < 0.05 and ^##^p < 0.01 indicate a statistically significant difference compared to Ti-M).

During cell mineralization, several osteogenesis-related proteins are expressed in the period of the osteogenic process. The expression of three important osteogenic proteins, which are bone sialoprotein (BSP), osteopontin (OPN) and osteocalcin (OCN) were analyzed using Western blotting, and the qualitative and quantitative results were shown in Fig. 6; the original electrophoresis blot images were shown in Fig. S4. The results showed that on day 7 of the incubation, the cells cultured on the anodized Ti2448-A1 and Ti2448-A2 exhibited greater OPN expression compared with the cells on the untreated Ti-M and Ti2448-M. The OPN protein is expressed during the early stage of osteogenic differentiation, and its expression decreased after day 14 of the incubation. In contrast, OCN, which is the protein expressed during the late stage of osteogenic differentiation, showed significant up-regulation when compared to the untreated group. Furthermore, at 21 days of incubation, the cells cultured on the anodized Ti2448-A1 and Ti2448-A2 expressed 2-fold greater OCN protein level than that of the untreated Ti-M and Ti2448-M. The cell mineralization and osteogenic protein expression results (Figs 5(o) and 6) showed that the anodized Ti2448-A1 and Ti2448-A2 were better able to induce hMSCs to initiate the osteogenesis process compared with the untreated Ti-M and Ti2448-M.

## Discussion

The long-term bio-corrosion resistance of Ti2448 alloy is crucial to its clinical applicability. This study presented a simple and rapid electrochemical anodization process that produced a hybrid layer with a dense inner oxide section capable of enhancing the bio-corrosion resistance of Ti2448 alloy (Figs 1(i) and (j) and 3).

It has been reported that microstructural feature, such as grain size, is important to the corrosion resistance of the materials^30^. As shown in Fig. S5, the optical microstructure observation showed that the Ti-M and Ti2448-M had apparent equiaxed grains with size of approximately 100 μm. On the other hand, a passive oxide layer on the surface of metallic materials has been shown to enhance corrosion resistance. In a report by Cheng *et al*., Ti2448 alloy releases fewer metal ions than CP-Ti and Ti6Al4V alloy in static immersion tests using AS, lactic acid solution, fluoridated saliva and fluoridated acidified saliva^15^. Bai *et al*. reported that Ti2448 alloy exhibits a wider passive region than CP-Ti and Ti6Al4V alloys, and its corrosion current density is comparable to that of CP-Ti in PBS, Hank’s solution and AS solution^16,17^. In this study, as shown in Fig. 3, Ti2448-M’s bio-corrosion resistance, in terms of E_corr_, was comparable to that of Ti-M in SBP and AS solutions. Furthermore, the anodized Ti2448 alloys showed much better bio-corrosion resistance, i.e., higher E_corr_ and lower I_pass_, than did Ti2448-M, due perhaps to the formation of a protective surface oxide layer. The passivity and corrosion resistance of various biomedical metals have been studied, showing that Ti, Nb, Zr and Sn metals exhibited great corrosion resistance in the following order: Nb > Ti > Zr > Sn^31,32^. Previous studies have reported that the addition of Nb and Zr improves the corrosion resistance of Ti alloys, by forming Nb_2_O_5_ and ZrO_2_, which strengthen the TiO_2_ oxide layer on Ti alloys^33–35^. This explains the good bio-corrosion resistance provided by surface oxides, containing mainly TiO_2_ and Nb_2_O_5_ with small amounts of ZrO_2_ and SnO, on Ti2448-M, Ti2448-A1 and Ti2448-A2.

Corrosion resistance also depends on the thickness and crystal structure of the oxide layer. The surface oxide layer that spontaneously forms on Ti and Ti alloys is less than approximately 10 nm^36^. Therefore, any surface treatments that can thicken this protective oxide layer were expected to improve the corrosion resistance of Ti and Ti alloys. Cigada *et al*. used anodic oxidation to improve the corrosion resistance of Ti6Al4V alloy in a buffered physiological solution by thickening the oxide layer^37^. Other research from Birch *et al*. and Velten *et al*. also showed that oxide layers thickened by thermal oxidation, anodic oxidation or sol-gel coating exhibit better corrosion resistance than that of polished Ti and Ti alloys^36,38^. In this study, the electrochemical anodization process significantly increased the thickness of the passive oxide layer on Ti2448-A1 and Ti2448-A2. Furthermore, the resulting oxide layer presented a hybrid structure, a mesoporous outer section and a dense inner section (thickness 50–120 nm) (Fig. 1(i) and (j)). The dense inner section was particularly effective in improving corrosion resistance, when evaluated in SBP and AS solutions. Furthermore, the crystal structure of the inner oxide layer was amorphous (Fig. 1(k)), i.e., lacking the grain boundaries required for electrochemical corrosion^39^. As illustrated by the polarization curves in Fig. 3, the anodized Ti2448 alloys with a thicker amorphous inner oxide layer displayed better bio-corrosion resistance, i.e., higher E_corr_, lower I_corr_, lower anodic and cathodic current densities, than did the untreated Ti2448-M. Our findings are consistent with those of previous studies, in which an amorphous oxide layer improves the corrosion resistance of metallic materials^39–41^. However, the results shown in Fig. 3 indicated that the anodized specimens presented lower cathodic breakdown potential on the cathodic side than did the untreated specimens (lower than approximately 170 mV in SBP solution; lower than approximately 170–300 mV in AS solution). This complex results pertaining to breakdown potential merit detailed discussion.

According to the previous reports by the co-authors of this study, the Ti2448 alloy, which possesses a low uniaxial tensile elastic modulus of approximately 45 GPa^13,14^, has properties that are beneficial for biomedical applications. The benefit of low elastic modulus of metal implant is to balance the inhomogeneous transfer of stress between the metal implant and the adjacent bone, and thus further prevents the stress-shielding effect. The proposed electrochemical anodization process produced a thin (50–120 nm) surface oxide layer (Fig. 1(i) and (j)), which would not affect the mechanical properties of bulk Ti2448 alloy. Therefore, the Ti2448 alloy with or without anodization treatment still had a uniaxial tensile elastic modulus of approximately 45 GPa.

For future clinical applications, bio-corrosion resistance of the Ti2448 alloy and good bio-interaction between the tissue and the Ti2448 alloy will both be of great concern. Numerous surface modification treatments have been developed to enhance the biocompatibility of Ti alloys. Micro-arc oxidation (MAO) treatment has been used to modify the surface topography and the chemical composition of Ti2448 alloy to improve the biological response^42–44^. However, the high voltages used in MAO treatment make the process relatively expensive and somewhat dangerous. The significantly improved bio-corrosion resistance and biocompatibility of Ti2448 alloy could be achieved at lower cost and more safely using the simple and rapid electrochemical anodization treatment proposed in this study.

It is commonly accepted that surface properties, such as topography, roughness, chemical composition and wettability, affect the biological responses at the bone-implant interface. Surface topography is a key property because it is able to regulate the response of cells on the surfaces of materials. In previous studies, nanoscale topographies, such as nanotubes, nanopits and nanonetworks, have influenced cell adhesion, spreading, migration, proliferation and differentiation *in vitro* as well as bonding strength and bone formation *in vivo*^45–48^. In this study, a disordered but homogeneous mesoscale porous oxide layer was created on the surface of Ti2448 alloys using electrochemical anodization treatment, and the chemistry of the oxide layer was similar among the test Ti2448 alloys. The pore sizes in the oxide layer ranged from a few nm to approximately 50 nm (Figs 1(c) and (d) and S1). Prolonged anodization under higher current density resulted in more pronounced anodic dissolution on Ti2448-A2 than on Ti2448-A1, leading to a larger surface pore size on Ti2448-A2. In terms of the surface feature reported by Rani *et al*.^49^, the mesofeatures of Ti2448-A1 were more similar to a mesoleaf, and the mesofeatures of Ti2448-A2 were more similar to a mesoscaffold. The surface free energy of the anodized Ti2448 alloys was enhanced, regardless of the difference in their surface mesofeatures. The results of the contact angle and the surface free energy analysis suggested that Ti2448-A1 and Ti2448-A2 had better surface wettability than Ti-M and Ti2448-M (Fig. 2). Bico *et al*. concluded that surface wettability is affected by the surface chemistry and topography^50^. In this study, the Ti2448-M, Ti2448-A1 and Ti2448-A2 specimens exhibited similar surface chemistries but different surface topographies. Therefore, the improved surface wettability caused by the electrochemical anodization treatment was mainly due to the formation of the mesoscale porous topography on the surface oxide layer. Cell attachment and spreading have been reported to be significantly greater on hydrophilic surfaces than on hydrophobic surfaces^51^. We expected that the hydrophilic surfaces of Ti2448-A1 and Ti2448-A2 would facilitate penetration by body fluids, thereby enhancing protein adsorption and triggering subsequent cell responses.

Protein adsorption occurs soon after the implantation of biomaterial within a biological environment, and is a key determinant of the responses of cells to the material surface. As shown in Fig. 4, the amount of albumin and fibronectin adsorbed on Ti2448-A1 and Ti2448-A2 was more than 1- to 3-fold greater than the amount adsorbed on Ti-M and Ti2448-M. In previous studies, one mechanism of protein adsorption is the electrostatic interaction between proteins and the material surface^52^. The isoelectric points of TiO_2_ and Nb_2_O_5_, two main components of the surface oxide layer on the anodized Ti2448 alloys, are approximately 4.1 to 4.5 and less than the pH of the physiological environment (~pH 7.0)^53,54^, thereby giving the oxide surface a negative charge. This negative charge attracts positively charged ions, such as Ca^2+^, which promote protein adsorption by acting as a bridge between the metal oxide surface and proteins.

Surface topography is another important factor influencing the adsorption of proteins. Sela *et al*. reported that the preferential adsorption of plasma protein can be attributed to an increase in the 3D surface area of modified Ti surface^55^. Richert *et al*. found that the greater surface area resulting from the creation of the nanoporous structure provides more binding sites for protein adsorption^56^. The mesoscale porous topography produced on anodized Ti2448 alloys presented a surface area far larger than that of untreated specimens, which greatly assisted in protein adsorption ability of the anodized Ti2448 alloys. Our findings are consistent with the above-mentioned studies. This behavior may trigger subsequent cell adhesion, proliferation, migration and differentiation^57–59^.

Previous researches have found that pro-adhesive proteins, such as fibronectin, trigger the formation of the focal adhesion complex by providing integrin binding sites for cell adhesion^59,60^. In the current study, the mesofeatures of Ti2448-A1 and Ti2448-A2 alloys led to a 1.5- to 2-fold increase in fibronectin adsorption, compared with Ti-M and Ti2448-M (Fig. 4). Additionally, the anodized specimens presented a higher concentration of the focal adhesion complex protein, vinculin, and more highly organized clustering (Figs 5(e–h) and S3(b)).

Macak *et al*. and Park *et al*. reported that a mesotube diameter of 15–30 nm is optimal for integrin clustering and the formation of focal adhesion complex^25,61^. Moreover, they also found that nanotubes with a diameter of 100 nm are unable to support the formation of focal adhesion complex, which ultimately leads to a significant increase in cell apoptosis. Pittrof *et al*. reported that cells cultured on mesotubes of small diameter are better able to settle down and begin actively producing extracellular matrix (ECM). The ECM anchoring made possible by mesotubes with a diameter of 15 nm appears to be crucial to advancing cell migration^62^. In this study, the surface mesoscale pore sizes of Ti2448-A1 and Ti2448-A2 ranged from a few nm to approximately 50 nm, which fall within the region suitable for cell adhesion and ECM production as well as for cell migration and proliferation. As shown in Figs 5(a–d) and S3(a), the lamellipodia of cells cultured on anodized specimens were far more extended than those cultured on Ti2448-M. This promoted the vinculin expression and actin arrangement, as shown in Fig. 5(e–h) and (i–l), respectively. Cell migration was also more pronounced on anodized specimens with mesoscale porous topography than on untreated specimens.

In this study, we failed to observe a significant difference in the number of cells on the various test specimens during the 7 days of incubation (Fig. 5(n)), which contradicts the findings of previous studies with regard to cell proliferation. This may be because the mesotopography of Ti2448 is a mixed mesoscale porous structure. Such non-periodic surface features may affect cell proliferation differently than the uniform structures that have been reported in previous studies. Nonetheless, Stein *et al*. described the relationship between proliferation and differentiation during the developmental sequence of rat osteoblasts^63^. They found an increase in alkaline phosphatase (ALP) expression immediately follows the proliferative phase^62^. This increase is followed by an increase in the expression of OPN and OCN at the start of mineralization (when cell proliferation is inhibited). In this study, we also analyzed the expression of osteogenic proteins and calcium deposition in ECM in order to gain a deeper understanding of how non-periodic mesosacle topography would affect the osteogenic differentiation and ECM mineralization of hMSCs. ECM mineralization (Fig. 5(o)) and OPN and OCN expression (Fig. 6) were more pronounced on anodized than on untreated specimens, which suggests that the cells on the anodized specimens proceeded from proliferation to differentiation more rapidly.

Topography- and integrin-mediated focal adhesions are critical inducers of downstream signaling in response to the characteristics of topography at the nanoscale^64,65^. Other studies have reported on the effects of nanoscale topography on promoting osteogenic differentiation and bone formation^66–70^. Those earlier results support our observation of increased vinculin expression and better cytoskeletal arrangement in Ti2448-A1 and Ti2448-A2 specimens, particularly in conjunction with an increase in calcium deposition and osteogenic protein expression. The above-mentioned studies reported the absorption of specific proteins with a considerable influence on the formation of integrin clusters. The formation of the clusters subsequently triggers the integrin-dependent signaling pathway via signaling molecules including focal adhesion kinase (FAK), extracellular signal-regulated kinase (ERK), protein kinase A (PKA) and Rho. Mechanotransduction is another mechanism regulating cell response and can be altered by the surface topography to mediate focal adhesion. This results in the transmission of signals into the nucleus to produce adaptive changes at the gene and protein levels. However, the mechanism underlying the altered function of hMSCs observed in this study requires further investigation.

**Figure 7:**
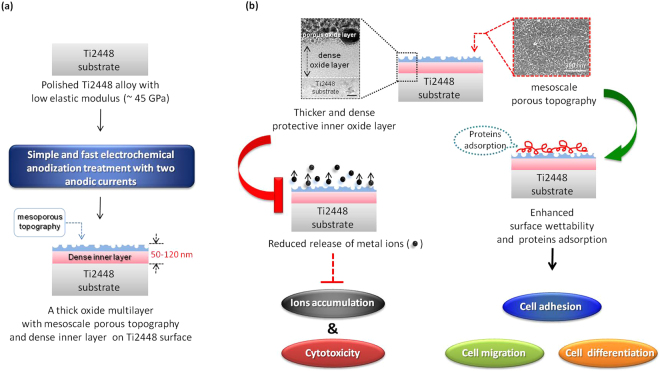
Illustration of how the hybrid surface oxide layer, produced by electrochemical anodization treatment, enhanced the bio-corrosion resistance and cell responses of the Ti2448 alloy: (**a**) a hybrid oxide layer with outer mesoporous topography and inner dense section could be produced using a simple and rapid electrochemical anodization treatment; (**b**) the thicker and dense inner section of the hybrid oxide layer reduced the release of metal ions. This decreased the potential risk of metal ion accumulation and cytotoxicity from the Ti248 alloy. Moreover, the outer mesoporous topography of the hybrid oxide layer improved the surface wettability and protein adsorption ability of the Ti2448 alloy. This enhanced the formation of the focal adhesion complex and cytoskeletal arrangement of bone cell, which, in turn, induced cell migration and osteogenic differentiation. The scale bar in the upper-left micrograph of (**b**) is 20 nm.

To summarize, this study demonstrated a simple and rapid electrochemical anodization process for the fabrication of a hybrid oxide layer, featuring a mesoporous outer section and a dense inner section, on the Ti2448 alloy, as shown in Fig. 7. This is the first study to prove that this unique hybrid surface structure significantly enhances the bio-corrosion resistance and bone cell responses of the newly developed Ti2448 alloy with a low uniaxial tensile elastic modulus of ~45 GPa. Future studies will investigate the effects of this hybrid surface structure on bone regeneration in an *in vivo* animal model, with the goal of benefiting bone implant applications.

## Conclusions

Using a simple and rapid electrochemical anodization treatment, a unique hybrid surface oxide layer, featuring a mesoporous outer section and a dense inner section, can be successfully produced on the surface of the newly developed β-type Ti-24Nb-4Zr-8Sn (Ti2448) alloy with a low uniaxial tensile elastic modulus of ~45 GPa. The dense inner section of the hybrid oxide layer enhances the resistance to bio-corrosion, as evidenced by an increase in the corrosion potential and a decrease in the anodic and cathodic current densities in both SBP and AS solutions. This dense inner section is expected to prevent ion release from the alloy substrate and decrease the risk of cytotoxicity caused by the accumulation of metal ions during implantations. Moreover, the mesoporous outer section of the hybrid oxide layer improves the surface wettability and have a range of pore sizes (from a few nm to approximately 50 nm) covering the scale of various types of proteins in the human body. These characteristics improve protein adsorption, thereby enhancing the formation of focal adhesion complexes and cytoskeletal arrangement. This, in turn, triggers signal transduction to induce cell migration and osteogenic differentiation. With these improvements in bio-corrosion resistance and bone cell responses, the proposed surface-modified Ti2448 alloys with a low uniaxial tensile elastic modulus have considerable potential for biomedical implant applications.

## Materials and Methods

### Specimen preparation

Discs (15 mm diameter and 1 mm thick) of β-type Ti2448 were used as substrates. The substrates were mechanically polished with SiC papers from #120 up to #1200 and then cleaned in ethyl alcohol. Pure commercial Ti discs (15 mm diameter and 1 mm thick) that were polished and cleaned according to the above-mentioned procedure were used as a reference control.

A potentiostat was used for the electrochemical anodization treatment to apply two different anodic currents, designated A1 and A2 (A1: 1 × 10^−6^ A/cm^2^; A2: 1.5 × 10^−6^ A/cm^2^), to the polished Ti2448 substrate for 8 and 12 min, respectively, in a 5 M NaOH solution at room temperature. The schematic illustration of electrochemical anodization treatment layout is shown in Fig. S6. The mechanically polished Ti and Ti2448 specimens that were not subjected to the electrochemical anodization treatment were termed Ti-M and Ti2448-M, respectively. The polished Ti2448 specimens treated by electrochemical anodization with the applied currents A1 and A2 were termed Ti2448-A1 and Ti2448-A2, respectively.

### Surface characteristics analysis

The surface morphology and average roughness of the test specimens were evaluated using field emission scanning electron microscopy (FE-SEM) and atomic force microscopy (AFM), respectively. Three FE-SEM images were used for analysis of the pore size and its distribution on the anodized Ti2448 specimens by using Image J software. The crystallographic structure and thickness of the outermost surface layer of the anodized Ti2448 specimens were evaluated using transmission electron microscopy (TEM). Prior to the TEM evaluation, the cross-sectional test specimens were prepared using a focused ion beam milling process. The chemical composition of the surface oxide layer of the specimens was analyzed by X-ray photoelectron spectroscopy (XPS). The surface wettability of the test specimens was analyzed using a contact angle goniometer, and the surface free energy of the specimens was calculated with two different solutions: polar double-distilled H_2_O and non-polar diiodomethane. The microstructural feature of Ti-M and Ti2448-M was observed using optical microscope and the grain size measurement was performed using a linear intercept method.

### Bio-corrosion resistance analysis

The surface corrosion resistance of the polished Ti-M and Ti2448-M and the anodized Ti2448-A1 and Ti2448-A2 was evaluated using a potentiostat. A saturated calomel electrode (SCE) and a Pt sheet were used as the reference and counter electrode, respectively. The test specimens with and without the electrochemical anodization treatment were used as the working electrodes. Neutral simulated blood plasma (SBP; pH 7.4)^71^ and modified Fusayama artificial saliva (AS; pH 5.2)^72^ were used as the corrosion test electrolytes and were maintained at 37 °C during the experiment. The SBP was used to simulate the environment inside the human body, and the AS was used to simulate that of the human mouth. All test specimens were placed into two different electrolytes, and the polarization curves of all specimens were measured from −1 V to +1.5 V (vs. SCE) with a scan rate of 1 mV/s. For calculating the current density of polarization curves, the projected area of the disc-shaped specimens (15 mm diameter) was used as the estimated area of the untreated specimens (Ti-M and Ti2448-M). However, the actual area of the anodized specimens (with a porous topography) was much larger than that of the untreated specimens. Thus, the surface areas of anodized specimens were calculated based on the pore size (refer to surface FE-SEM images in Fig. 1(c) and (d)), the thickness of the outer porous section (refer to cross-sectional TEM images in Fig. 1(i) and (j)), and pore size distribution (refer to pore size vs. distribution in Fig. S1). The area ratios of the anodized Ti2448-A1 and Ti2448-A2 to Ti2448-M were approximately 6.0 and 6.5, respectively.

### Protein adsorption analysis

Two different types of protein were used as model proteins to evaluate the difference in protein adsorption on the test specimens. One protein was bovine serum albumin (BSA, Sigma), and albumin is the most abundant protein in human plasma^73^; the other protein was fibronectin (Sigma), which is an important protein involved in cell adhesion, migration, proliferation and differentiation^74^. The test specimens were immersed in two different phosphate buffered saline (PBS) solutions: one containing 5 mg/ml BSA and the other containing 50 μg/ml fibronectin. After 5 min of incubation at 37 °C, the test specimens were washed with double-distilled H_2_O and were dried at room temperature. XPS was used to analyze the nitrogen spectra (in terms of N1s) of the test specimens to estimate the ability for proteins to adsorb on the various treated test specimens.

### Cell culture

Human bone marrow mesenchymal stem cells (hMSCs) were harvested and isolated as described in previously published research^75^. The hMSCs were cultured in Dulbecco’s modified Eagle medium (DMEM, Sigma) containing 10% fetal bovine serum (FBS, Biological Industries). The purified hMSCs were then transfected using a retrovirus with the gene for green fluorescence protein (GFP), and those transduced cells were termed hMSCs-GFP and were cultured in RPMI-1640 (Sigma) supplemented with 5% FBS and 10% horse serum (Invitrogen)^76^.

### Cell adhesion morphology analysis

The hMSCs-GFP were seeded on the test specimens at a density of 2 × 10^4^ cells/cm^2^. After 1 h of incubation, the *in situ* cell distribution of the hMSCs-GFP was observed using a fluorescence microscope, and the cells were then fixed in 4% paraformaldehyde and 2% glutaraldehyde. After fixation, all test specimens were dehydrated in a graded series of double-distilled water containing 10% to 100% ethanol, followed by critical point drying. The test specimens were coated with platinum, and the cell adhesion morphology was observed and imaged using FE-SEM. Three random FE-SEM images were taken from each of three independent specimens for each test group, and the cell spreading area of each single cell was measured using Image J software.

Furthermore, the expression of focal adhesion complex related protein and the cytoskeletal arrangement were estimated by immunofluorescence staining. The hMSCs were seeded on the specimens at a density of 2 × 10^4^ cells/cm^2^. After 6 h of incubation, the cells were fixed in 4% paraformaldehyde and treated with 0.3% Triton X-100 in PBS. Then, the cells were incubated with mouse monoclonal anti-vinculin (Sigma) at 4 °C overnight followed by incubation in a secondary antibody conjugated with fluorescein for 2 h (Chemicon). The nuclei and F-actin were stained with diamidino-2-phenylindole (DAPI, Sigma) and rhodamine phalloidin (Cell Signaling), respectively. Both the expression of focal adhesion complex related protein, vinculin, and the cytoskeletal arrangement were observed and imaged using a fluorescence microscope. Five random images were taken from each of three independent specimens for each test group, and the number of vinculin-positive focal adhesion complex formation in each single cell was counted using Image J software.

### Cell migration analysis

A wound-healing assay was used to estimate the cell migration on the test specimens. The hMSCs-GFP were seeded at a density of 5 × 10^4^ cells/cm^2^. After 24 h of incubation, a gap of approximately 250 μm was scratched on the surface of the test specimens, which were then incubated for 6 h. During the cell incubation, the cell migration was observed and imaged using a fluorescence microscope at the time when the gap was scratched (0 h) and again 6 h afterwards.

### Cell proliferation analysis

A 3-(4.5-dimethylthiazol-2-yl)-2,5-diphenyl tetrazolium bromide (MTT) assay was used to evaluate the cell proliferation. The hMSCs cells were seeded on the test specimens at a density of 1 × 10^4^ cells/cm^2^ for 1, 4 and 7 days. The MTT reagent was added at the above-mentioned time points and incubated at 37 °C for 4 h. After the MTT treatment, the formazan was dissolved by isopropanol, and the absorbance was measured using a microplate photometer at 570 nm. A higher optical density (OD) represented a greater number of cells. The cell culture set-up and the detailed procedure of MTT assay were shown in Fig. S7.

### Cell mineralization analysis

Alizarin Red S staining was used to evaluate the deposition of calcium-containing compounds during the initial cell differentiation. The hMSCs were seeded on the test specimens at a density of 1 × 10^4^ cells/cm^2^ for 7, 14 and 21 days. For this evaluation, after one day of incubation, the culture medium was changed to osteogenic differentiation medium consisting of DMEM supplemented with 50 µg/ml ascorbic-2 phosphate (Sigma), 10 mM β-glycerophosphate (Sigma) and 10^−8^ M dexamethasone (Sigma) every two days. The cells that attached on the surface of the test specimens were fixed with 70% ice-cold ethanol and stained with Alizarin Red S staining reagent. After staining, a 10% cetylpyridinium chloride (CPC, Sigma) solution was added to the stained test specimens, and the absorbance was measured at 540 nm using a microplate photometer.

### Cell differentiation analysis: osteogenic protein expression

Western blotting was used to analyze the expression of osteogenic proteins during cell differentiation. The hMSCs were seeded on the test specimens at a density of 1 × 10^4^ cells/cm^2^, and the total cell lysate was harvested after 7, 14 and 21 days of cell incubation using a radioimmunoprecipitation assay (RIPA) buffer. Then, 20 µg of the total cell lysate was separated using 10% polyacrylamide gel electrophoresis, transferred onto nitrocellulose membranes and was then blocked with 5% BSA/PBS. After blocking, the membranes were incubated with specific primary antibodies, including anti-bone sialoprotein (anti-BSP, Millipore), anti-osteopontin (anti-OPN, Abcam), anti-osteocalcin (anti-OCN, Abcam) and anti-GAPDH (Abcam); the membranes were then incubated with secondary antibodies. The secondary antibodies used for this evaluation were goat-anti-rabbit IgG-HRP (Abcam) and rabbit-anti-mouse IgG-HRP (Abcam). An ECL detection kit (Millipore) was used as the substrate to measure the specific protein expression with HRP on immunoblots, and images were obtained using a luminescence/fluorescence image system (LAS4000, Fujifilm). The relative photographic density was quantified using the Multi Gauge V2.2 software.

### Statistical analysis

The number of specimens was 3 for all measurements, and the results were expressed as the means ± standard deviation (SD). Student’s two-tailed t-test was used to determine the level of the significance, and *p* < 0.05 was considered statistically significant.

## Electronic supplementary material

Supplementary Figures S1-S7
