# Supplementary material for: A unique hybrid-structured surface produced by rapid electrochemical anodization enhances bio-corrosion resistance and bone cell responses of β-type Ti-24Nb-4Zr-8Sn alloy
Source: Sci Rep. 2018 Apr 26;8:6623. doi: 10.1038/s41598-018-24590-x (PMC5920132; doi:10.1038/s41598-018-24590-x)
Supplement: Supplementary file 1 — Supplementary Figures S1-S7 [file 41598_2018_24590_MOESM1_ESM.doc]

**Supplementary Information**

**A unique hybrid-structured surface produced by rapid electrochemical anodization enhances bio-corrosion resistance and bone cell responses of β-type Ti-24Nb-4Zr-8Sn alloy**

Chia-Fei Liu, Tzu-Hsin Lee, Jeng-Fen Liu, Wen-Tao Hou, Shu-Jun Li, Yu-Lin Hao, Haobo Pan, and Her-Hsiung Huang*

**Figure S1. Surface pore size and its distribution, in terms of occurrence rate (%), on anodized Ti2448 specimens.** (a) Ti2448-A1; (b) Ti2448-A2.

**Figure S2. XPS depth profiling data, in terms of atomic concentration (%), of test specimens’ surfaces.** (a) Ti-M; (b) Ti2448-M; (c) Ti2448-A1; (d) Ti2448-A2.

**Figure S3. (a) Quantitative analysis of relative cell spreading area (vs. Ti-M) of hMSC-GFP cultured on the surface of test specimens for 1 h; (b) Quantitative analysis of focal adheison complex number (per cell) of hMSCs cultured on the surface of test specimens for 6 h.** * p<0.05 and ** p<0.01 indicate a statistically significant difference compared to Ti2448-M; # p<0.05 and ## p<0.01 indicate a statistically significant difference compared to Ti-M.

**
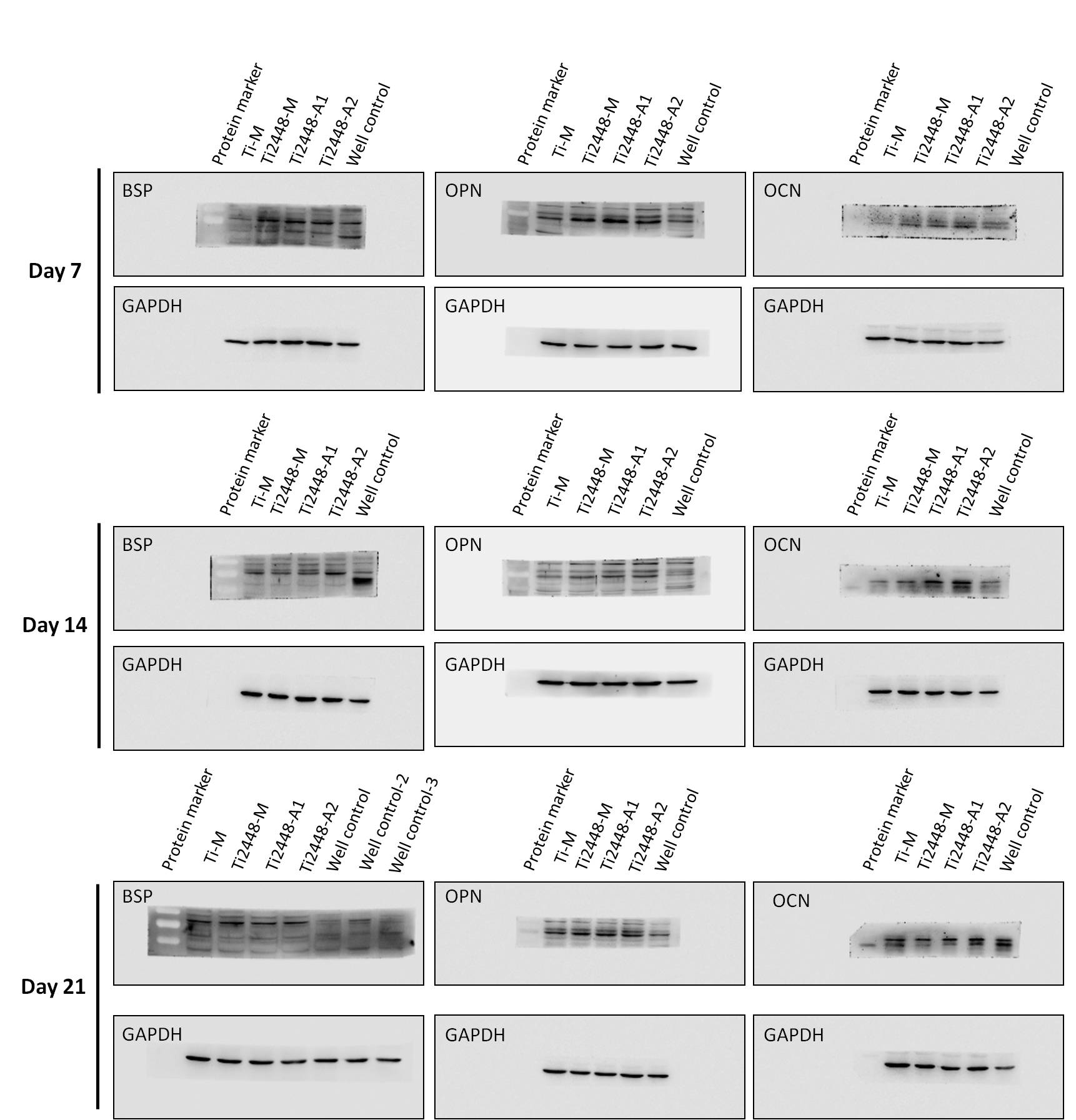
**

**Figure S4. Original electrophoresis blot images of osteogenic protein marker expression of the hMSCs cultured on the test Ti and Ti2448 specimens for 7, 14 and 21 days.**

**
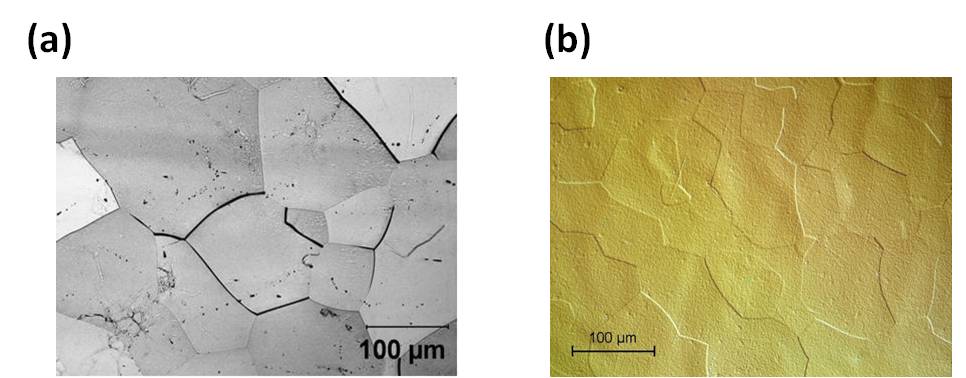
**

**Figure S5. Microstructural images observed using optical microscope.** (a) Ti-M and (b) Ti2448-M.

**Figure S6. Schematic illustration of electrochemical anodization treatment layout.** The saturated calomel electrode (SCE) was used as reference electrode (RE), the Ti2448 specimen as anode (working electrode, WE) and the platinum (Pt) as cathode (counter electrode, CE). The distance between cathode and anode was approximately 5 mm. The electrolyte used in this study was 5M NaOH solution and contained in a Pyrex glass.

**Figure S7.** **Schematic illustration of MTT assay process ((a)-(e)) for cell proliferation analysis.** (a) Seeding cell suspension on the surface of test specimen (one test specimen per well of 12-well tissue culture plate); (b) After 2 h of incubation at 37℃, adding fresh cell culture medium to the culture well in order to make the specimen fully immersed in the culture medium. The culture medium was refreshed every two days; (c) At every time point (1, 4 and 7 days), the culture medium was removed and the MTT reagent was added into culture well to produce formazan on the test specimen; (d) After 4 h of incubation at 37°C, the test specimen was transferred to the new culture well; (e) Adding isopropanol to dissolve the formazan on the test specimen for the following absorbance measurement.
